# Oxidative stress assessment in intestine of newborn rats submitted to hypoxia and reoxygenation with tadalafil^1^

**DOI:** 10.1590/s0102-865020190040000007

**Published:** 2019-04-29

**Authors:** Luciana Cristina Teixeira, Renato Toshio Murashita Fujiki, Caio Vinicius Coitinho Paiva de Souza, Camila Mendes de Souza, Maysa Moreira Mismetti, Ricardo Artigiane-Neto, Edna Frasson de Souza Montero, José Luiz Martins

**Affiliations:** I Fellow Master degree, Postgraduate Program in Interdisciplinary Surgical Science, Universidade Federal de São Paulo (UNIFESP), Brazil. Technical procedures; acquisition, analysis and interpretation of data; manuscript preparation.; II Graduate student, UNIFESP, Sao Paulo-SP, Brazil. Technical procedures; acquisition, analysis and interpretation of data; manuscript preparation.; III Graduate student, Faculdade de Medicina Santa Marcelina, Sao Paulo-SP, Brazil. Technical procedures; acquisition, analysis and interpretation of data; manuscript preparation.; IV Associate Professor, Head, Department of Pathology, UNIFESP, Sao Paulo-SP, Brazil. Histopathological examinations, critical revision, final approval.; V Associate Professor, Surgical Gastroenterology Division, Department of Surgery, UNIFESP, Sao Paulo-SP, Brazil. Scientific and intellectual content of the study, critical revision, final approval.; VI Full Professor, Pediatric Surgery Division, UNIFESP, Sao Paulo-SP, Brazil. Scientific, intellectual, conception and design of the study; critical revision; final approval.

**Keywords:** Hypoxia, Enterocolitis, Necrotizing, Tadalafil, Rats

## Abstract

**Purpose:**

To evaluate the functional and structural response of tadalafil effects in the intestinal mucosa, using an experimental model of hypoxia and reoxygenation injury in rats.

**Methods:**

The animals were divided into 4 groups: CTL, H/R, H/R+Td and M+Td. The newborn rats allocated in groups H/R, H/R+Td and M+Td were submitted twice a day, to a gas chamber with CO_2_ at 100% for 10 minutes and afterward reoxygenation with O_2_ at 98% for 10 minutes, in the three first days of life. Tadalafil dose was given to newborn of group H/R+Td and to the pregnant rat of group M+Td. Histological analysis was made with hematoxylin-eosin technique and oxidative stress through nitrite and nitrate levels and lipid peroxidation.

**Results:**

The histological analysis showed a reduction of mucosa alterations in the groups that received tadalafil. In the oxidative stress evaluation, occurred an increase of NO levels and less lipidic peroxidation in the ileum segments that received tadalafil.

**Conclusion:**

Tadalafil provides tissue protection when administered independently to both, pregnant or newborns.

## Introduction

 The necrotizing enterocolitis (NEC) is one of the most common gastrointestinal emergencies in newborn^1^, its main risk factor is the prematurity, which associated to formula feeding, leading to bacterial invasion and triggering intestinal ischemia/ reperfusion phenomena (H/R)^2^. It is estimated that the disease affects between 5% and 15% of premature and about 7% of term infants admitted into neonatal intensive care units and that the mortality rate ranges between 18% and 45%, depending on the prematurity degree^1^.

 The etiopathogeneses of NEC remains uncertain in such a way that effective measures in the treatment and prevention are still a challenge. Theories suggest that the lack of intestinal perfusion in newborns would cause a perturbation in the intestinal mucosa still immature jointly with a series of inflammatory reactions in cascade, with local invasion and bacterial proliferation, which would result in coagulation necrosis of affected areas^1^. As from these hypotheses, is assumed that studies directed to initial events of inflammatory cascade caused by H/R are more likely to provide favorable data in the search for effective prophylaxis. The search for substances that protect the intestinal mucosa against the consequences of H/R is important^2^.

 Tadalafil is part of the family of selective inhibitors of type 5 phosphodiesterase enzyme responsible for the degradation of GMPc, a drug commonly used for treatment of erectile dysfunction and that has been studied in the prevention of several other diseases, among them: cardiovascular^3^, stem cells^4^, pulmonary hypertension^5^, uterine growth restriction^6^, acute renal failure^7^, and hepatic lesions from ischemia and reperfusion^8^. The studies demonstrated the beneficial effects of tadalafil in microcirculation regulation and reduction of damages caused by H/R lesions.

 The tadalafil mechanism of action occurs in nerve endings and endothelial cells, which release NO, responsible for stimulating the enzyme guanylate cyclase, which converts guanosine triphosphate (GTP) into guanosine cyclic monophosphate (GMPc)^8^. The elevated GMPc rates generate a reduction of calcium concentration, reduction of muscle contraction force, relaxation of a musculature consequent vasodilatation with a drop in blood pressure^3^. The type 5 phosphodiesterase (PDE-5) isaniso enzyme that acts in the degradation of GMPc transforming it into inactive GMP. The inhibition of PDE-5 promotes an increase in the intracellular concentrations of GMPc extending its physiologic action^8^. This increase in levels of GMPc causes relaxation of vascular smooth muscle, which improves the supply of blood microcirculation and inhibition of platelet aggregation, preventing the obstruction of small vessels^9^.

 For being a highly selective therapy in the inhibition of PDE-5 that presents half-life much longer(17h) that other drugs from the same family such as sildenafil and vardenafil (4-8h), tadalafil is a promising clinical alternative, once allows a longer lasting effect with fewer daily doses. However, more studies are necessary to describe the risks and benefits from its use^4^. The effects of tadalafil on dysfunctions and structural intestinal injuries caused by H/R are not fully explained. This research has as purpose to test the action of tadalafil in intestinal mucosa of rats and assess its effect in tissue injury caused by H/R.

## Methods

 All procedures performed in the current study were evaluated and approved by the Ethics Committee for Animal Use - UNIFESP, under number 2739041117.

 Four pregnant rats were used from Wistar strain, which were maintained in an assisted environment: temperature from 21-23ºC, bright/ dark cycle of 12h, adequate accommodation, hygiene, the ration for the species and water at will. After birth, the newborns were maintained with the mother for breast milk feeding.

### Experimental design

 The pregnant rats and their respective offspring were distributed randomly in 4 study groups: control group (CTL), newborn animals submitted only to hypoxia-reoxygenation (H/R), newborn animals submitted to pre-treatment with tadalafil 30 minutes before the hypoxia-reoxygenation (H/R + Td) and pregnant rat receiving a pre-treatment with tadalafil 24 hours before delivery and offspring submitted to hypoxia-reoxygenation (M + Td).

 In the newborn animals of group H/R + Td, 30 minutes before the first, third and fifth events of H/R, with an intraperitoneal insulin needle, tadalafil solution (5mg/kg i.p) in solution 0.175% DMSO. In pregnant rats of group M+Td oral medication was provided, by gavage, in aqueous solution (10mg/kg), 24 hours before delivery.

 The newborn rats from groups H/R, H/R +Td e M + Td were placed in a special transparent acrylic chamber for controlled gas inhalation. They were submitted to hypoxia with CO_2_ at 100% for 10 minutes (verifying the existence or not of hypothermia) and, afterward reoxygenation with O_2_ at 98% for another 10 minutes, twice a day on the three first days of life, all being weighed at the end. This protocol was described by Özkan *et al.* ^10^. After the events, the offspring were placed again into the company of the mother at the appropriate temperature. 

 On the 4^th^ day of life, the offspring of all groups were submitted to intraperitoneal anesthesia with xylazine (8 to 10 mg/kg) and ketamine (60 to 80 mg/kg). Laparotomy was performed and searched for macroscopic signs of ischemic-edema lesion, distension, perforation, necrosis, or hemorrhage in the viscera. The ileum was totally removed in separated segments that were fixed and conserved in buffered formalin at 10%, dehydrated in alcohol 70%, and mounted in paraffin blocks. All offspring of the experiment were submitted to euthanasia with cervical dislocation on the fourth day under anesthesia, after collecting the materials for study.

### Histopathological analysis

 Fragments of 1 cm of ileum were isolated and separated from each animal. The samples for histological analysis were fixed in buffered formalin, dehydrated, embedded in a paraffin block, with 3µm cuts. Afterward, mounted on slides, with part stained by hematoxylin-eosin (HE) for the morphological exam (mucosa, submucosa, vessels, and muscle fibers). The samples for oxidative stress assessment were isolated and stored in cryogenic tubes in liquid nitrogen and transferred afterward to the freezer at temperatures below -30ºC.

 An optical binocular microscope was used with 100- and 400-times magnification to examine the slides stained by the hematoxylin-eosin method and intestinal morphology of ileum samples was classified according to hydropic degeneration degree. Fragments of ileum were classified according to the degree of hydropic degeneration from 0 to 2: Grade 0: intact mucosa without alterations; Grade 1: mucosa with well-constituted villi, but with a small number of hydropic degeneration signs; Grade 2: mucosa with a high degree of hydropic degeneration.

### Oxidative stress

 The oxidative stress was evaluated as from the determination of tissue NO, as from nitrite and nitrate analysis, and dosage of malondialdehyde (MDA). Tissue NO was determined by Griess method^12^. A solution (1:1) containing sulfanilamide at 1% in H_3_PO_4_ a 5% and naphthyl-ethylenediamine (Sigma) at 0.1% was added 100µL of homogenate (100mg of tissue in 1.0mL of PBS, pH 7.4) and the absorbance at 546 nm was measured using a spectrophotometer. The nitrite, one of the stable metabolites of NO, was then estimated by comparison with a standard curve built with NaNO_2_. The total protein of homogenate was quantified by Lowry method *al*.^12^ and absorbance compared to albumin standard. The quantification of NO was expressed in µg per mg of total protein. The Ohkawa *et al*.^13^ method was used to dose the MDA, in order to evaluate the lipidic peroxidation found in the lesioned tissue.

### Statistics

 The Chi-square test was applied for analysis of results for confrontation the histological evaluation results and variance analysis of Kruskal-Wallis to study possible differences regarding NO and MDA. The level of significance was set at 0.05. 

## Results

### Histopathological analysis

**Figure 1:**
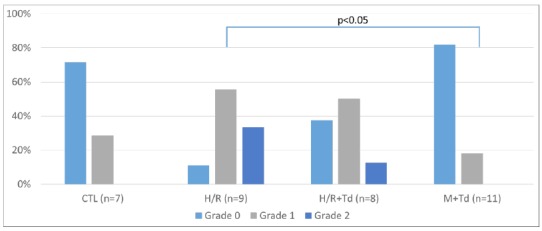
Distribution among groups regarding the hydropic degeneration of the ileum enterocytes.

**Figure 2:**
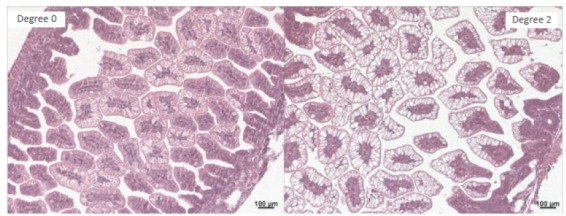
On the left, photomicrography of normal ileum considered the absence of hydropic degeneration (Control Group - Grade 0) and on the right considered hydropic degeneration (H/R Group - Grade 2) HE.

 Ileal segments showed a different response to hypoxia/ reoxygenation when tadalafil was used, decreasing the grade of hydropic degeneration. H/R group = 89% of Grade 1+2; H/R+Td=88% and M+Td=100% of Grade 0 +1; M+Td<H/R, p<0.05 (Figs. 1 and 2). 

### Nitrite/Nitrate and MDA

**Figure 3:**
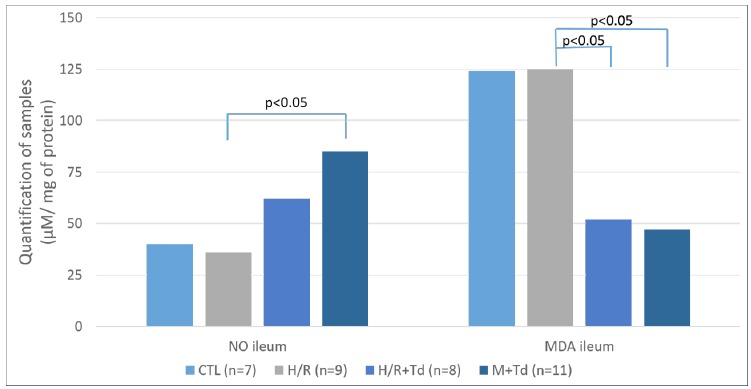
Ileal concentration of nitrite/nitrate (NO) and malondialdehyde (MDA) according to groups.

 The assessment of oxidative stress in ileum was made through quantification of MDA and nitrite/nitrate (NO). Ileal samples: the following results, expressed as mean ±SD, were obtained for NO (CTL=44±14; H/R=41±14; H/R+Tad=65±24; M+Tad=88±33); and MDA (CTL=138±41; H/R=141±46; H/R+Tad=50±14; M+Tad=54±16) (Fig. 3).

## Discussion

 Based on the results obtained tadalafil could be a promising drug in the treatment and prevention of H/R damage, especially when provided during the prenatal period.

 However, even though the drug has demonstrated to be effective, the histological evaluation did not show lesion compatible with the damages that occur in NEC, as in our previous studies^14^ ^-^ ^16^. The hydropic degeneration degree and ballooning of enterocytes may be a precursor lesion of necrosis, corresponding to an initial stage of the injury proposed previously^17^ ^,^ ^18^. 

 Gomes *et al.* ^16^ had to move to another experimental model^10^, augmenting the time of hypoxia exposition, to obtain a similar degree of lesion obtained by Cintra *et al.* ^15^. This protocol^10^, already consolidated, which reproduces the issue of oxidative stress and intestinal mucosa injury, it was not enough to get the same morphological alterations.

 It was thought the higher weight of the newborns justified that difference. Therefore, in the present study, it was used in the same model. However, pups were heavier, weighing 5.0 to 7.0 grams (data not shown), than those (4.2 to 6.0 grams). The mildest lesions may also be related to the diet of newborns, which includes breast milk, an important protective factor for intestinal mucosa^19^.

 In the evaluation of oxidative stress, an increase in the levels of NO was expected, once the drug would interfere with its pathway, causing vasodilatation. But in the quantification of MDA, a reduction of its values was expected for reflecting the lipidic peroxidation degree caused by H/R events^20^

^^The results obtained demonstrate that tadalafil showed to be effective in the protection of ileum against oxidative stress, more evident in the group where the mother rat received the drug, although the intervention in the newborn also demonstrates certain protection degree.

 Therefore, new studies are necessary to better elaborate the reproduction method of lesion characteristic of ENC as well as to apply for its promisor role in the prevention and treatment in the newborn environment, providing improvement for the patients’ life quality.

## Conclusion

 Tadalafil provides tissue protection, independent of those given for pregnant or in newborns. 
